# Health Systems and Sustainability: Doctors and Consumers Differ on Threats and Solutions

**DOI:** 10.1371/journal.pone.0019222

**Published:** 2011-04-27

**Authors:** Jane Robertson, Emily J. Walkom, David A. Henry

**Affiliations:** 1 Clinical Pharmacology, School of Medicine and Public Health, The University of Newcastle, Callaghan, New South Wales, Australia; 2 Institute for Clinical Evaluative Sciences, Toronto, Canada; Erasmus University Rotterdam, The Netherlands

## Abstract

**Background:**

Healthcare systems face the problem of insufficient resources to meet the needs of ageing populations and increasing demands for access to new treatments. It is unclear whether doctors and consumers agree on the main challenges to health system sustainability.

**Methodology:**

We conducted a mail survey of Australian doctors (specialists and general practitioners) and a computer assisted telephone interview (CATI) of consumers to determine their views on contributors to increasing health care costs, rationing of services and involvement in health resource allocation decisions. Differences in responses are reported as odds ratios (OR) and 99% confidence intervals (CI).

**Results:**

Of 2948 doctors, 1139 (38.6%) responded; 533 of 826 consumers responded (64.5% response). Doctors were more concerned than consumers with the effects of an ageing population (OR 3.0; 99% CI 1.7, 5.4), and costs of new drugs and technologies (OR 5.1; CI 3.3, 8.0), but less likely to consider pharmaceutical promotional activities as a cost driver (OR 0.29, CI 0.22, 0.39). Doctors were more likely than consumers to view ‘community demand’ for new technologies as a major cost driver, (OR 1.6; 1.2, 2.2), but less likely to attribute increased costs to patients failing to take responsibility for their own health (OR 0.35; 0.24, 0.49). Like doctors, the majority of consumers saw a need for public consultation in decisions about funding for new treatments.

**Conclusions:**

Australian doctors and consumers hold different views on the sustainability of the healthcare system, and a number of key issues relating to costs, cost drivers, roles and responsibilities. Doctors recognise their dual responsibility to patients and society, see an important role for physicians in influencing resource allocation, and acknowledge their lack of skills in assessing treatments of marginal value. Consumers recognise cost pressures on the health system, but express willingness to be involved in health care decision making.

## Introduction

Healthcare systems in both developed and emerging economies face the problem of insufficient resources to meet the anticipated health care needs of all citizens. Ageing populations, longer life expectancy, increasing demand for services, new technologies and new medicines all contribute to the financial pressures. Doctors and patients (consumers) are aware of these pressures [1]. To those working in health services these pressures lead to implicit or explicit rationing [2]; patients experience these effects as waiting lists for services and limited access to some medicines or procedures in the public health system [3].

The moral dimensions of healthcare rationing have been widely debated [2], [4], [5]. Some argue that a doctor's sole responsibility is to his or her own patients (patient advocacy role) [6]–[8], giving primacy to the patient's needs over possible social concerns about inequity. Others see the physician's responsibility as encompassing the efficient use of medical resources (public interest advocacy) as well as the interests of the individual patient [5], [9]. This sense of sharing responsibility for the management of scarce resources by physicians, other experts and consumers has been posited as “protecting the medical commons” [10], [11]. While there are calls for the medical profession to engage more in improving systems of care and population health, neither medical education nor the practice environment has fostered this [3], [12], [13].

Pressures in the Australian health care system mirror those in other settings. Concerns about unsustainable rises in health care costs have been flagged in a series of intergenerational health reports [14]. All Australians have access to publicly subsidised treatment in a public hospital, which is free at the point of care for everyone. Medicare also subsidises community-based treatment by general practitioners and specialists [15]. Where practitioners ‘bulk bill’ Medicare for their fees the patient does not pay an additional co-payment. Patients cannot access specialists directly – they must be referred by a GP, so these professional groups have a co-dependent rather than competitive relationship. In 2007–8, 53 percent of the Australian population purchased some level of private health insurance [16]. This is a voluntary option (with tax incentives), and supplements the services available through Medicare. The type of insurance varies, and covers some costs in private hospitals and services such as private dental, optical, and ambulance services. Access to pharmaceuticals is via the taxpayer funded Pharmaceutical Benefits Scheme (PBS) [17]. Patients pay a fixed amount or co-payment for each prescription, the amount depending on their welfare status. A ‘safety net’ cuts in with heavily discounted prescriptions after a certain number have been dispensed in a calendar year.

Australian doctors work with this complex mix of publicly and privately funded services. It is unclear to what extent Australian physicians recognise these issues and see a role for themselves in improving the efficient management of the health care system. We also know little about the Australian general public's opinions about health care spending [18]. In a climate of concern about rising health care costs, the media are drawing attention to the apparently insatiable demand for services, challenging whether patient expectations are realistic, and whether current models of service delivery are sustainable [19]–[21].

In view of the paucity of data on the views of doctors about their roles in a financially constrained system, we aimed to compare doctors' and consumers' perceptions of the Australian health care system and measure their concerns about rising health care costs. We wished to explore their views on factors that contribute to increasing health care costs and where the responsibilities lie for managing these costs. Specifically, we wished to probe their views about the need to restrict access to health care services (‘rationing’) and who should be involved in such decisions. Finally, given their different roles in the health care system, we wanted to examine whether the views of GPs and specialists differed on these issues.

## Methods

### Ethics statement

The surveys and the project were approved by the University of Newcastle Human Research Ethics Committee and the Hunter Area Research Ethics Committee. In the case of the participating doctors (mail survey), return of the completed questionnaire was accepted as informed consent. Consumer participants in the study were randomly selected from the Electronic White Pages (telephone directory) and were sent pre-notification letters describing the study. Consumers gave verbal consent to participate at the time of the telephone interview; this consent was recorded by the Hunter Valley Research Foundation (HVRF) who administered the questionnaires on behalf of the researchers. Verbal consent is a standard procedure used by the HVRF based on extensive experience conducting telephone surveys of this type; requiring written consent produces low response rates and survey samples unlikely to be representative of the study population. Methods of recruitment and for obtaining consent were approved by the two Ethics Committees.

### Survey development

The underlying concepts of costs and responsibility for managing costs were derived from the literature [5]–[9], [18], [22]–[24]. Items for the doctor mail survey and the Computer Assisted Telephone Interviews (CATI) of consumers were developed from instruments used in other studies exploring respondents' views on health care cost issues [18], [25], [26]. We included questions about factors we believed to be relevant in the Australian setting. In addition, we conducted two focus groups with local medical practitioners (one with five general practitioners, the second with eight specialists). Transcripts of the focus group were coded for themes; these themes were used to guide the development of survey items.

### Survey questions

As far as possible we used the same items and response scales for the doctor and consumer surveys (24 items common to both), making minor modifications to the wording of a small number of terms in the consumer survey only to account for different levels of knowledge of medical terminology and concepts. Direct comparisons between doctors and the public were confined to those items.

#### Questions common to the surveys of doctors and consumers

Attitudes towards the health care system in Australia: 1 item assessing the quality of health care services (5-point scale *Very poor* to *very adequate*) and 1 item assessing concern about the costs of health care (4-point scale C*oncerned* to *Not at all concerned*). Possible causes of increasing health care costs: 14 items (4-point Likert scale *No contribution* to *Major contribution*). Attitudes towards managing health care costs, the responsibilities of doctors, allocating health care resources: 7 items scored using a 5-point Likert scale *Strongly disagree* to *Strongly agree*.

Respondents in both surveys were asked to indicate whether they would be willing to be involved in deciding how health dollars should be spent. If they were not willing, they were asked to nominate who they would prefer to make these decisions on their behalf.

#### Consumer only questions

Awareness of health care costs (*Decreasing*, *Increasing*, *Staying the same*), and actions being taken to the control these costs (*Too little*, *About the right amount*, *Too much*).

We recorded the gender and age (18–44 years, 45–59 years, ≥60 years) of respondents, private health insurance and concession card status. The latter two categories are not mutually exclusive: some low income families choose to purchase additional private insurance to avoid public hospital waiting lists and to access private hospital services (27). We examined responses for the consumers by age, gender, private insurance and concession card status.

#### Doctor only questions

For the doctor only questions, we compared responses for GPs and specialists. One question sought views on the ease of access to health care services (5-point Likert scale *Very poor* to *very adequate*); 5 questions addressed the contribution of new technological advances and practice issues to increasing health care costs (4-point Likert scale *No contribution* to *Major contribution*); 12 items sought agreement with statements about health care costs and their influence on medical practice; and 6 items focussed on rationing and cost containment (5-point Likert scale *Strongly disagree* to *Strongly agree*). We recorded the gender, practice location, medical specialty and years since graduation for these doctors and compared these demographic characteristics for mail survey respondents and non-respondents and with the overall commercial database from which the sample was chosen.

A two-phase pilot of the doctor mail survey was conducted; the first with 24 GPs and specialists; the revised survey, which included minor wording changes, was administered to a further five GPs and specialists. A three-phase pilot of 30 interviews was conducted for the consumer CATI. Minor modifications were made to the interview script to clarify the meaning of several questions for consumers and these were tested in subsequent phases of the pilot.

Copies of the doctor and consumer surveys are available on request.

### Participants and Recruitment

#### Doctor survey

A list, comprising a random sample of 1500 General Practitioners (GPs) and 1500 specialists was obtained from a commercial database of over 54,000 practising doctors (AMPCo Direct, http://www.ampcodirect.com.au). The specialists were chosen from 13 specialties in proportion to their gender, specialty and State representation in the database. The anonymous survey was distributed by mail in May 2006, with a follow-up survey sent to all participants three weeks later.

#### Consumer survey

The Hunter Valley Research Foundation administered the survey using a Computer Assisted Telephone Interview (CATI) system. Households in New South Wales (NSW), Australia were randomly selected from the Electronic White Pages. Pre-notification letters were sent to the selected households describing the aims and methods of the study. A minimum of 6 call attempts were made to contact each household, and once contact had been made, at least another 5 attempts were made to speak to the respondent to obtain either a completed interview or a refusal. Respondents were aged 18 years or over and randomly selected based on age relative to other householders (e.g. youngest, second oldest). The survey took approximately 20 minutes; interviews were completed between April and June 2006.

### Analysis

Descriptive statistics (proportions) were used to summarise the data. Responses for each item are reported separately. Likert scale responses were dichotomised into *major or moderate* contributor to costs (vs no contribution or little contribution) and *agree and strongly agree* (vs strongly disagree, disagree or neither agree nor disagree) in order to compare responses between doctors (specialists plus GPs combined) and consumers, and between specialists and GPs for questions only asked of the doctors. Differences between groups are reported as odds ratios (OR), with the consumers as reference group for the doctor versus consumer comparisons and GPs as the reference group for the specialist versus GP comparisons. Because we performed multiple comparisons we were conservative in our calculations and report here the Odds Ratios with their 99% confidence intervals.

## Results

### Response rates

#### Doctor survey

1139 responses were received from 2948 eligible contacts; the 52 ineligible contacts included envelopes returned to sender, retired/deceased doctors, person unavailable for duration of survey, giving a survey response rate of 38.6%. After exclusion of incomplete survey forms, 1118 doctors (514 GPs, 604 specialists) were included in this analysis. There were no statistically significant differences in the distributions of specialty, state and gender between survey respondents, the mail-out sample, or in the overall database of practising doctors from which the sample was drawn (data not displayed). This suggested our survey respondents were generally representative of GPs and specialists in Australia.

#### Consumer survey

A total of 533 interviews were completed from 826 eligible contacts giving a response rate of 64.5%. Among respondents, 37.5% were male; ages ranged from 18 to 89 years (mean 52 years); 58% had private health insurance and 44.8% reported holding a health care concession card. Rates of private health insurance were comparable to those reported for the overall Australian population in the year of the study [27].

### General views on health care services in Australia

Doctors were much more likely than consumers to rate the quality of health care services provided to Australians as *adequate or very adequate* (81.2% doctors, 40.2% consumers; OR 6.4, 99% CI 4.7, 8.8). Specialists were more likely than GPs to rate access to these services as *adequate or very adequate* (64.9% vs 56.8%; OR 1.4, 99% CI 1.0, 2.0).

Most consumers (91%) recognised health care costs were increasing, with 76% responding that too little was being done to control them.

The costs of providing health care services were an issue for both doctors and consumers; doctors were less likely than consumers to choose the highest level of concern on the response scales (26.2% vs 85.7%; OR 0.17, 99% CI 0.13, 0.23), however doctors were more likely than consumers to respond they were *fairly concerned* about costs (52.4% vs 26.2%; OR 5.0, 99% CI 3.6, 7.0). Consumer concerns about costs were age-related, with those aged ≥60 years more likely to respond *concerned* than those aged <60 years (78.2% vs 62.4%; OR 2.2, 99% CI 1.2, 3.9).

### Views on causes of increasing health care costs (see Table 1)

**Table 1:** Moderate or major contributors to increasing health care costs - Doctors (specialists and GPs combined) versus consumers.

	n (%) stating a major or moderate contributor to heath care costs		
	DoctorsN = 1118	ConsumersN = 533	Odds ratio(99% CI)*
**Population characteristics**			
Ageing population	1079 (96.5)	481 (90.2)	2.99 (1.67, 5.42)
More people with chronic illnesses	987 (88.3)	454 (85.2)	1.31 (0.87, 1.96)
**New advances and practice issues**			
Development of expensive new medicines and interventions	1055 (94.4)	408 (76.5)	5.13 (3.32, 8.01)
Availability of high-tech medical equipment and procedures	978 (87.5)	411 (77.1)	2.07 (1.44, 2.98)
Practice of defensive medicine for fear of litigation	900 (80.5)	408 (76.5)	1.26 (0.90, 1.76)
New treatments for cancer	734 (65.7)	350 (65.7)	0.99 (0.75, 1.34)
**Social context**			
Community expectations for access to the latest technologies	857 (76.7)	357 (67.0)	1.62 (1.19, 2.20)
People not taking responsibility to keep themselves healthy	751 (67.2)	456 (85.6)	0.35 (0.24, 0.49)
Decreases in informal care (e.g. by family and friends)	603 (53.9)	346 (64.9)	0.63 (0.47, 0.84)
Doctors' reluctance to refuse patient requests for tests, drugs	590 (52.8)	281 (52.7)	1.00 (0.76, 1.32)
Patients expecting a test or prescription at every doctor's visit	555 (49.6)	343 (64.4)	0.55 (0.41, 0.73)
**External pressures**			
Lobby group and public pressure to fund particular diseases	546 (48.8)	320 (60.0)	0.64 (0.48, 0.84)
Drug company advertising to persuade people to ask for medicines (in newspapers, television current affairs)	420 (37.6)	351 (65.9)	0.31 (0.23, 0.42)
Drug company promotions to doctors to prescribe medicines	410 (36.7)	354 (66.4)	0.29 (0.22, 0.39)

*Consumers are the reference category.

#### Population characteristics

Doctors were generally more likely to identify an ageing population and more people with chronic illness as a cause of increasing health care costs than consumers, although the difference was only statistically significant for the question about the role of ageing populations (96.5% vs 90.2%; OR 3.0, 99% CI 1.7, 5.4).

#### New advances and practice issues

Doctors were significantly more concerned than consumers about the costs of new medicines and interventions, and availability of high-tech equipment and procedures than consumers (OR 5.1, 99% CI 3.3, 8.0 and OR 2.1, 99% CI 1.4, 3.0 respectively). While there was a trend for doctors to be more concerned than consumers about the costs associated with the practice of defensive medicine for fear of litigation, this difference was not statistically significantly different (OR 1.3, 99% CI 0.90, 1.8). Two-thirds of both doctors and consumers were concerned about the costs of new treatments for cancer as a major or moderate contributor to increasing health care costs.

#### Social context

Doctors were significantly more likely than consumers to see community expectations of access to the latest technologies as a major or moderate contributor to increasing health care costs (76.7% vs 67%; OR 1.6, 99% CI 1.2, 2.2). However, doctors were less likely than consumers to attribute increasing health care costs to people not taking responsibility for keeping themselves healthy (67.2% vs 85.6%; OR 0.35, 99%CI 0.24, 0.49), patients expecting a test or prescription at every visit (49.6% vs 64.4%; OR 0.55, 99%CI 0.41, 0.73), and to lower levels of social support (informal care) in the community (53.9% vs 64.9%; OR 0.63, 99% CI 0.47, 0.84). Sixty-one percent of consumers were concerned about the cost implications of patients wasting medicines by hoarding or filling repeat prescriptions but not using the medicine; this last question was not asked of doctors.

#### External pressures

Doctors were much less likely than consumers to report that they considered the following to be important cost drivers: external pressures from drug company promotion to doctors (36.7% vs 66.4%; OR 0.29, 99% CI 0.22, 0.39), drug company advertising to consumers (37.6% vs 65.9%; OR 0.31, 99% CI 0.23, 0.42), and the activities of lobby groups and public pressure to fund particular diseases (48.8% vs 60%; OR 0.64, 99% CI 0.48, 0.84).

### Attitudes towards managing health care costs (see Table 2)

**Table 2:** Attitudes towards health care costs and rationing - Doctors (specialists and GPs combined) versus consumers.

	n (%) agreeing or strongly agreeing with statement		
	DoctorsN = 1118	ConsumersN = 533	Odds ratio(99% CI)*
**Attitudes towards health care costs**			
Patients should pay a greater part of the health care bill so they will be more cost-conscious	538 (48.1)	169 (31.7)	2.00 (1.50, 2.68)
The *Government* should educate the public about the true costs of health care	958 (85.7)	495 (92.9)	0.46 (0.27, 0.75)
*Doctors* should educate their patients about the true costs of health care	534 (47.8)	395 (74.1)	0.32 (0.23, 0.43)
Only the treating physician and the patient should decide if a treatment is “worth the cost”	333 (29.8)	360 (67.5)	0.20 (0.15, 0.27)
Not matter how small the chance of benefit, the physician should offer a medical treatment regardless of the cost	316 (28.3)	442 (82.9)	0.08 (0.06, 0.11)
It is *not* the doctor's responsibility to be concerned about the costs of health care to society	156 (14.0)	185 (34.7)	0.31 (0.22, 0.42)
**Rationing and health care resource allocation**			
The public should be consulted about rationing decisions and allocation of health care resources	784 (70.1)	375 (70.4)	0.99 (0.73, 1.34)

*Consumers are the reference category.

Doctors and consumers differed significantly in their attitudes towards managing health care costs. More doctors than consumers agreed that patients should be required to pay a greater share of their health care costs to increase their cost-consciousness (48.1% vs 31.7%; OR 2.0, 99% CI 1.5, 2.7). Both doctors and consumers identified a need for educating the public about the costs of health care; although the great majority of doctors and consumers saw a role for Government in this process, this view was less common among doctors (85.7% vs 92.9%; OR 0.45, 99% CI 0.27, 0.75). Fewer doctors than consumers saw a role for the medical professions in educating the public (47.8% vs 74.1%; OR 0.32, 99% CI 0.23, 0.43). Doctors were significantly less likely than consumers to consider that only the treating doctor and patient should decide whether a treatment is worth the cost (30% vs 67.5%; OR 0.20, 99% CI 0.15, 0.27), or that a treatment should be offered regardless of how high the cost, and how small the benefit, of treatment might be (28.3% vs 82.9%; OR 0.08, 99% CI 0.06, 0.11).

Similar proportions (70%) of doctors and consumers identified a role for public consultation about rationing decisions and allocation of health care resources.

While one-third of consumers agreed that it is *not* the doctors' responsibility to be concerned about health care costs to society, 89.8% of consumer respondents agreed that doctors have an obligation not to waste the money that taxpayers have provided for health care. A minority (35.5%) of consumers considered that the cost of a medical treatment should be considered only when the patient must pay all or part of the cost. Around one half of consumers (51.4%) agreed that the Government has a duty to provide the money necessary to meet all the health needs of the population.

### Specialists compared to GPs

#### Views on causes of increasing health care costs

Specialist doctors and general practitioners held broadly similar views on issues relating to waste, duplication and inefficiency that might influence costs in the health care system (see Table 3). However, specialists were significantly less likely than GPs to see waste in the public hospital system as a cost driver (44% vs 57.6%; OR 0.58, 99% CI 0.42, 0.80).

**Table 3:** Moderate or major contributors to increasing health care costs - Specialists versus GPs.

	n (%) stating a major or moderate contributor to heath care costs		
	SpecialistsN = 604	GPsN = 514	Odds ratio(99% CI)*
**New advances and practice issues**			
Increases in standard diagnostic tests (routine tests, investigations)	474 (78.5)	431 (83.9)	0.70 (0.46, 1.06)
Interventions that offer minimal benefits for their cost	397 (65.7)	304 (59.1)	1.32 (0.96, 1.84)
Duplication of tests, investigations (GPs/specialists/hospitals)	292 (48.3)	255 (49.6)	0.95 (0.69, 1.30)
Wasting of resources in the public hospital system	266 (44.0)	296 (57.6)	0.58 (0.42, 0.80)
Uncapped budgets (fee-for-service for GPs, private practice)	228 (37.7)	164 (31.9)	1.29 (0.93, 1.81)

*GPs are the reference category.

#### Managing health care costs, rationing and cost containment

There were few differences between specialist doctors and GPs on issues concerning management of costs in the health care system (see Table 4). However, more specialists than GPs considered that physicians need more training in recognising and identifying marginally beneficial services and that the present remuneration system provides no incentive to be cost conscious (OR 1.4, 99% CI 1.0, 2.0 and OR 1.6, 99% CI 1.2, 2.2 respectively). Additionally, specialists were less likely than GPs to report prescribing or ordering tests because they felt the need to be seen to be doing something for the patient (15.2% vs 21.8%; OR 0.64, 99% CI 0.43, 0.97).

**Table 4:** Attitudes towards health care costs and rationing - Specialists versus GPs.

	n (%) agreeing or strongly agreeing with statement		
	SpecialistsN = 604	GPsN = 514	Odds ratio(99% CI)*
**Attitudes towards health care costs and influence on medical practice**			
As individual clinicians, physicians should play a role in helping to control health care costs	407 (67.4)	327 (63.6)	1.18 (0.85, 1.65)
My concerns about the social cost of health care do not change my behaviour when treating individual patients	260 (43.0)	233 (45.3)	0.91 (0.66, 1.25)
My only responsibility is the care of my patient, regardless of the cost	185 (30.6)	152 (29.6)	1.05 (0.74, 1.49)
When deciding how to treat a patient, I think about other uses of the health care money (*opportunity cost*)	155 (25.7)	148 (28.8)	0.85 (0.60, 1.22)
It is reasonable to consider cost when the test, drug or intervention is likely to be only of marginal benefit	539 (89.2)	452 (87.9)	1.14 (0.69, 1.88)
Physicians need more training in recognizing and identifying marginally beneficial services	363 (60.1)	264 (51.4)	1.43 (1.04, 1.96)
Undergraduate and intern training programs should include sessions on cost-effective medical practices and prescribing	457 (75.7)	363 (70.6)	1.29 (0.90, 1.85)
Cost-effectiveness information alone is probably not enough to persuade me to change my practice patterns	361 (59.8)	279 (54.3)	1.25 (0.91, 1.72)
I sometimes prescribe or order tests because I feel the need to be seen to be doing something for the patient	92 (15.2)	112 (21.8)	0.64 (0.43, 0.97)
I am indifferent to drug costs	52 (8.6)	37 (7.2)	1.21 (0.67, 2.24)
A PBS listing means the Government has assessed the drug as being cost-effective (‘value-for-money’).	190 (31.5)	172 (33.5)	0.91 (0.65, 1.28)
Under present remuneration system there is no incentive to be cost conscious	351 (58.1)	237 (46.1)	1.62 (1.18, 2.23)
**Rationing and cost containment**			
There is a legitimate need for cost containment in today's health care environment	522 (86.4)	432 (84.0)	1.21 (0.77, 1.90)
Rationing decisions are an inevitable part of medicine	457 (75.7)	378 (73.5)	1.12 (0.78, 1.61)
Costs to society should always be considered when making clinical decisions	233 (38.6)	208 (40.5)	0.92 (0.67, 1.28)
Medical people have a role in administration to influence the allocation of health care resources	559 (92.5)	461 (89.7)	1.43 (0.81, 2.53)
I would be willing to implement rationing decisions made by groups informed by doctors	344 (57.0)	263 (51.2)	1.26 (0.92, 1.74)
I would be willing to implement rationing decisions made by government authorities	182 (30.1)	132 (25.7)	1.25 (0.88, 1.78)

*GPs are the reference category.

Around two thirds of specialists and GPs recognised a role for their profession in helping to control health care costs and only 30% of both groups held that their sole responsibility was the care of the patient before them. However, for around 44% of the doctor respondents, concern about the societal costs of health was reported not to influence the management of individual patients. For the majority of doctors (60% specialists, 54% GPs) cost-effectiveness information alone was not sufficient to make them change their practices. Yet, 88% of each group agreed that it is reasonable to consider costs when the test, drug or intervention was likely to be of marginal value. Around one-third of respondents agreed that a PBS listing for a medicine meant that it had been assessed as cost-effective (a “value-for-money” choice).

There were no significant differences between specialists and GPs in their expressed attitudes towards rationing and cost containment. Most notably, both groups of doctors were strongly of the view that physicians have a role in health administration with the intent of influencing the allocation of health care resources (92.5% specialists, 89.7% GPs).

### Health care resource allocation

Consumers were more likely than doctors to indicate a personal willingness to have a role in deciding how health dollars should be spent (57% consumers, 43.2% specialists, 29.6% GPs p<0.0001). Consumers who were unwilling to be personally involved nominated doctors (39.7%) or other health professionals (17.9%), politicians or government representatives (30.6%), or consumer groups (16.6%) as having a role in making rationing decisions on their behalf. Of 717 doctors who reported being unwilling to be involved in resource allocation decisions, 91.5% nominated other doctors as first or second preference with 66% ranking health economists as first or second preference. Consumers and politicians were ranked first or second preference for only 16.2% and 7.7% respectively.

## Discussion

Both doctors and consumers believe that the health care system is under pressure because of rising costs; but there were striking differences between the two groups on what was contributing to these problems and how, and by whom, they should be managed.

Overall, doctors appeared less concerned than consumers about the threat posed by increasing costs to the sustainability of the healthcare system. However, they were more concerned than consumers with the effects of an ageing population, the consequential high prevalence of chronic diseases, and the related costs of new drugs and new technologies. They were also more likely than consumers to view ‘community demand’ for new technologies as a major cost driver. Doctors were more likely than consumers to consider that patients should be expected to pay a greater share of healthcare costs.

In contrast, consumers were more likely than doctors to attribute rising costs to people not maintaining their own health, expecting a prescription at every visit, and there being less social support generally in the community than previously. Consumers were more likely than doctors to identify the adverse effects of pharmaceutical industry promotion of their products. Consumers were more likely to consider that decisions about expensive treatments should be left to doctors and their patients, and also to consider that treatment should be offered irrespective of its cost. Like doctors, the majority saw a need for public consultation when decisions were made about new treatments, but they were more likely to report a willingness to be involved in such decisions. Both doctors and consumers identified the need for better education of the public about health care costs, although differed in who should provide this education.

This study did not assess doctors' awareness of the cost implications of their own decisions. Most doctors recognised increases in the use of standard diagnostic tests as a contributor to costs, however it is unclear whether this was viewed as overutilisation (of tests, procedures and specialist visits) that has been described in other settings [28]. Not only are there cost implications of more tests and early treatment intervention, tests may lead to incidental findings and unnecessary anxiety, and subsequent diagnostic labels can lead to treatments where the benefits of early intervention are unclear [29].

### Attitudes of respondents

It seems that consumers tend to judge other consumers more harshly than do doctors. While consumers may see themselves as responsible medicine and health service users [30], they appear to be less certain about the behaviours of others, seeing irresponsible and wasteful practices as contributors to increasing health care costs. US consumers have likewise recognised their contribution to wasteful healthcare spending associated with obesity, smoking and poor adherence to drug regimens, compounded by behaviours such as going to emergency rooms for non-emergency care, and demands for costly treatments and technologies [31].

Doctors were more inclined to identify the ageing in the community and increasing prevalence of chronic diseases, and the availability of new treatments, as major cost drivers. But they were not free of subjective judgments. They identified patient expectations of access to new services as a substantial contributor to increasing costs. This is not a new suggestion, and perceptions influence actions. Doctors' opinions about patient expectations for a medicine have been shown to be a strong determinant of prescribing [32]. While this survey cannot address the question of whether the perceptions of patient demand for new technologies are well-founded, there is evidence that doctors tend to overestimate patient expectations [33]. When patients are fully informed about treatment options and given help with decision-making, demand for some types of interventions, including surgery has been shown to decrease [34]. However, patients with complex and serious illnesses generally wish to delegate authority for decision-making about treatments and procedures to their doctor [32], [35].

Consumers expressed stronger views than doctors about the impact of the pharmaceutical industry on professional practices. The available evidence shows that some types of industry contact have adverse effects on prescribing [36]. In an interesting parallel to consumers' views of themselves and others, doctors tend to acknowledge the influence of industry on their peers but not themselves.

While the views of consumers and doctors are probably subjective, a number of the concerns they expressed mirror those identified by Government as drivers of increasing health care costs. The 2010 Intergenerational Report [14] used projections on past spending patterns to foreshadow future cost pressures from the interaction of an ageing population and increasing demand for health services and the funding of new technologies. These were reflected in growth of costs of hospitals, medical services and pharmaceuticals due to an ageing population, and an increased prevalence of chronic diseases. The Report also identified growth in spending on residential aged care as a driver of future health care costs, noting this will be influenced by the mix between residential care and care in the community.

### The reality of rationing

Most doctors in this survey recognised rationing as an inevitable feature of medical practice. This is consistent with evidence from surveys conducted in a variety of settings [37]. While this may be accepted as inevitable by health care workers, consumers tend to see rationing in terms of failure of politicians to increase funding to meet health care needs [1]. In this study, just over half of the consumers suggested Government should provide the money needed to meet all of the health care needs of the population.

### Responses to increasing costs

While acknowledging the need for restraint, respondents to this study were unclear about how rationing should be applied. Doctors were more inclined to give weight to decisions informed by the opinions of other doctors than those made only by government authorities. However, they acknowledged the importance of engaging the public in rationing decisions. Others have suggested that this “administrative gatekeeping” [38] may be the most ethical response to rationing for doctors, allowing them to implement fair cost-reducing guidelines passed at higher levels within the health care system, while at the same time avoiding having to make rationing decisions in individual cases. As in an earlier Australian study [18], the consumers in this study expressed willingness to participate in such resource allocation decisions.

But within the doctor-patient relationship saying no is hard, and complicated by the interdependence, respect, concern and affection in this relationship [39]. This may pose the greatest challenge to GPs who have ongoing relationships with patients and their families and is reflected in responses in this survey – more GPs than specialists in this survey felt that no matter how small the chance of benefit and regardless of the cost, the physician should offer all treatment options to patients. However, a consequence of this position of trust is that the GP is well positioned to improve the quality of decisions for patients [34].

Consumers responding to this survey felt that doctors should tell patients about medical treatments regardless of costs and how small the likely benefits. However, knowing about treatments they cannot afford or service shortages that directly affect them may distress patients [40]–[42]. The dilemma for doctors is that failing to tell patients about all treatment options might be viewed as patronising and as a betrayal of trust, and not acting in the patient's best interest [43]. If rationing is used it should be overt rather than covert with cost considerations explicit, transparent and consistent [13].

### Responses at a societal level

Although respondents to our surveys recognised the importance of public involvement in decisions about health resource allocation, there is no agreement in the literature about the best approaches for doing this [44], [45]. Italian developments include the use of citizen juries to deliberate on health care decisions like prostate cancer screening [46], [47]. Public participation in discussions of policy options requires knowledge and understanding of the complexity of the health care system [48]. Some have argued a role for the public in advising on the allocation of health care resources but not for rationing decisions [4].

Mostly, discussions about rationing tend to focus on how to assess the value of new technologies [49]; less often discussed is the notion of ‘disinvestment’ in health care technologies [13], and the reduced use or elimination of potentially wasteful practices [50]. However, this requires knowledge of, or an ability to identify, treatments of marginal value; doctors in this study identified a need for greater skills in this area. In the face of increasing pressures on health care financing, educating physicians to be cost aware is important. Some argue it is a critical responsibility of medical schools and residency programs to provide this training; the challenge being how to get ‘low’ real-world concerns such as costs into medical school curricula dominated by ‘high knowledge’ including molecular biology and genomics [13], [51]. Doctors in this survey reported the need for more training in assessing marginal services but even provided with information on cost-effectiveness many said they were unlikely to change their practices. Sessions and Detsky [52] have recently proposed a framework for such medical education to equip doctors for their dual roles as patient advocates and allocators of resources – a core course examining economic influences on clinical decisions and practical application of the principles in clinical and residency curricula.

Making rational choices relies on good information on comparative effectiveness and cost-effectiveness. Medical journal articles and reviews should be an important source of information. A recent review [53] highlights some difficulties. Fewer than one-third (31.7%) of studies evaluating medications published in six high impact general and internal medicine journals (June 2008 and September 2009) were comparative studies and few focused on safety and cost issues. Many cost-effectiveness studies are flawed and industry funding appears to be an important source of bias [54]. This underscores the importance of impartial sources of information such as the guidance provided by health technology assessment bodies like the National Institute for Health and Clinical Excellence (NICE) in the UK and the Canadian Agency for Drugs and Technologies in Health (CADTH).

In an environment of concern about increasing health care costs there are opportunities for governments, health policy makers and medical educators to inform the debate about health spending. Government has a role in making costs and the limits of health care funding more visible to both doctors and consumers. Given the expressed willingness of some doctors and consumers to have a role in resource allocation decisions, policy makers need to find ways to incorporate the views of doctors and consumers in decision-making. Informed citizen juries currently being evaluated in a number of countries may provide a useful model for consumer engagement. As some doctors have indicated a need for further training about economics, medical educators should consider the development of undergraduate and postgraduate programs to meet this need. These alone will not manage the problems of finite resources and increasing demand, however they will contribute to a more informed and inclusive debate about future health funding.

### Study limitations

The study has a number of limitations. We asked consumers to focus on health care policy not their personal experiences of health care and we avoided reference to specific clinical conditions or treatments. Responses may have differed for chronic conditions amenable to medical management compared to life-threatening diseases. A criticism might be that consumers don't know how the health care system works so can't comment on sources of increasing health care costs. However, their concerns about the costs of new technologies mirrored those of doctors.

A potential criticism of this study is the different response rates in the surveys of doctors and consumers. The higher response rate from consumers was achieved by random digit dialling. While males were under-represented among the consumer respondents, this is a common feature of telephone surveys of this type [55]. As Australia has a parallel public private funding system for health it could be argued that insurance status could have influenced responses. However, rates of private health insurance in our respondents were comparable to those in the Australian population [27] and we found no differences in responses based on this factor. It was not feasible to conduct a survey of doctors using random digit dialling and we only had postal addresses for the doctors selected in the sample. While the response rate from the doctors' survey was low, it is comparable to response rates to mail surveys by medical practitioners [56] and other mail surveys of GPs and specialists conducted by us [57], [58]. Importantly, there were no statistically significant differences in the distribution by specialty, state and gender between survey respondents and non-respondents, and all doctors in the commercial AMPCo database from which the sample was drawn, the responses should be generally representative of GPs and specialists in Australia.

A major strength of this study is that doctor and consumer surveys were conducted over a comparable period of time. Although the survey methods that we used differed for the doctors and consumers, the techniques were appropriate and likely to maximise the response rates in each case. There was sufficient common material to enable us to perform valid comparisons of the two responding groups.

### Conclusion

In Australia, doctors and consumers appear to hold different views on the sustainability of the healthcare system, and a number of key issues relating to costs, cost drivers, roles and responsibilities. Doctors recognise their dual responsibility to patients and to society, see an important role for physicians in health care administration to be able to influence resource allocation, and acknowledge a lack of training in assessing treatments of marginal value. Consumers recognise cost pressures on the health system and express willingness to be involved in health care decision making.
